# Prognostic Value of Volume-Based Positron Emission Tomography/Computed Tomography in Nasopharyngeal Carcinoma Patients after Comprehensive Therapy

**DOI:** 10.1155/2018/1384281

**Published:** 2018-02-21

**Authors:** Yueli Tian, Khamis Hassan Bakari, Shanshan Liao, Xiaotian Xia, Xun Sun, Chunxia Qin, Yongxue Zhang, Xiaoli Lan

**Affiliations:** ^1^Department of Nuclear Medicine, Union Hospital, Tongji Medical College, Huazhong University of Science and Technology, Wuhan 430022, China; ^2^Hubei Key Laboratory of Molecular Imaging, Union Hospital, Tongji Medical College, Huazhong University of Science and Technology, Wuhan 430022, China

## Abstract

**Objective:**

We assessed the prognostic value of standardized uptake value (SUV) and volume-based methods including whole-body metabolic tumor volume (WBMTV) and whole-body total lesion glycolysis (WBTLG) using ^18^F-fluorodeoxyglucose positron emission tomography/computed tomography (PET/CT) of patients with nasopharyngeal carcinoma (NPC) after therapy.

**Methods:**

A total of 221 posttherapy NPC cases were enrolled, all of whom had undergone PET/CT scanning and follow-up in this retrospective study. The diagnostic results of PET/CT were analyzed and compared with histopathological diagnosis or clinical follow-up. Receiver operator characteristic curves, the Kaplan-Meier method, and the log-rank test were used to assess the optimal cutoff values for WBMTV and WBTLG to identify independent predictors of survival.

**Results:**

The detection rates of the threshold SUV were 2.5, 20%, and 40%, and SUV background methods were 65.6% (378/576), 80.2% (462/576), 71.5% (412/576), and 90.4% (521/576), respectively (*P* < 0.005). Patients with a WBMTV < 8.10 and/or a WBTLG < 35.58 had significantly better 5-year overall survival than those above the cutoffs (90.7% versus 51.2%, *P* < 0.001; 91.7% versus 50.4%, *P* < 0.001), respectively. Multivariate Cox regression modeling showed both WBTLG (RR, 1.002; *P* = 0.004) and age (RR, 1.046; *P* = 0.006) could be used to predict overall survival. WBTLG (RR, 1.003; *P* < 0.001) may have predictive relevance in estimating disease-free survival.

**Conclusions:**

SUV volume-based threshold background methodology had a significantly higher detection rate for metastatic lesions. WBTLG could be used as an independent prognostic indicator for posttherapy NPC.

## 1. Introduction

Nasopharyngeal carcinoma (NPC) is one of the commonest epithelial-derived malignant tumors of the head and neck, and it is associated with the highest incidence of nodal and/or distant metastases [1–3]. As a chemo- and radiosensitive tumor, the current standard treatment for locally advanced NPC is concurrent chemoradiotherapy [4, 5], which can result in 5-year survival and disease-free rates up to 70% [6, 7]. However, recurrence of the disease may occur [7, 8]. Known prognostic factors of NPC include a history of smoking, TNM stage classification, clinical and molecular prognostic variables, and elevated plasma Epstein–Barr virus DNA [9]. Clinically, none of them can accurately define the prognosis of NPC patients.

^18^F-Fluorodeoxyglucose positron emission tomography/computed tomography (^18^F-FDG PET/CT) is suitable for the evaluation of various types of cancers including NPC. Being noninvasive, the tool has the ability to visualize and measure physiological function and biochemical processes (metabolism) of most cancers and has been widely used in the diagnosis, staging, management, monitoring of treatment response, and recurrence detection of many malignancies [10]. ^18^F-FDG PET parameters that are used as independent prognostic factors during or after chemotherapy and radiotherapy include maximum standardized uptake value (SUVmax), metabolic tumor volume (MTV), and total lesion glycolysis (TLG) [11–13]. Multiple reports have shown that SUVmax, defined as the maximum SUV in a region of interest (ROI) containing the tumor, is one prognostic factor in some cancers [11, 14, 15]. In addition to SUVmax, MTV and TLG have also been widely used as tumor metabolic and volumetric parameters in ^18^F-FDG PET/CT. The volume of tumor tissue with active ^18^F-FDG uptake is defined as MTV [16–18]. TLG is the median SUV in an ROI multiplied by the MTV. The utilization of MTV and TLG is crucial in dealing with metabolically active lesions and tumor invasiveness [19].

Different thresholds such as the absolute, relative, and background relative thresholds have been frequently applied in lesion segmentation but it is still a contentious matter as to which threshold should be designated for segmenting the volume of lesion. Incongruent results of the prognostic importance of SUVmax, MTV, and TLG in NPC patients have been reported by previous studies [20, 21]. The importance of volumetric ^18^F-FDG PET parameters has not been fully assessed, thus making it difficult to accurately identify the best predictors of treatment outcome. The aim of this study was to investigate the prognostic value of WBMTV and WBTLG obtained from ^18^F-FDG PET/CT images in NPC patients after comprehensive therapy. Four different threshold methods were assessed and an optimal threshold for segmentation of recurrent metastatic lesions was selected.

## 2. Patients and Methods

### 2.1. Patients

Permission to conduct this study was granted by the institution review board of Tongji Medical College, Huazhong University of Science and Technology, and informed consent was obtained from all patients. A total of 221 patients (169 males [76.4%] and 52 females [23.5%]; median age 46 ± 12 y, range: 17–75 y) were retrospectively analyzed. All patients had performed ^18^F-FDG PET/CT imaging in the PET Center of Union Hospital, Tongji Medical College of Huazhong University of Science and Technology, between September 2003 and May 2013 after treatment. All patients had received surgery and/or definitive intensity-modulated radiotherapy (IMRT) and/or adjuvant platinum-based chemotherapy. Inclusion criteria were as follows: (1) histologically proven nasopharyngeal carcinoma; (2) complete clinical and imaging data; (3) the patients receiving therapy before the PET/CT scan. Exclusion criteria were as follows: (1) diabetes and pregnancy; (2) absence of other malignant lesions or borderline lesions. Follow-up time was 34.4 ± 24.8 months (range: 5–120 months) and ended in October 2013. The time of death in patients signifies the endpoint of follow-up.

### 2.2. ^18^F-FDG PET/CT Imaging

All patients fasted for at least 6 h before the examination and were injected intravenously with 3.74 MBq (0.10 mCi)/Kg ^18^F-FDG. All patients had their blood glucose concentration measured and established to be <6.6 mmol/L. Patients then rested for 45–60 minutes in a quiet, dark environment and later drank 300–500 mL of water to empty their bladder before PET/CT scanning. ^18^F-FDG PET imaging was performed using a Discovery LS PET/CT system (GE Medical Systems). A CT scan was acquired for attenuation correction using the following parameters: a tube voltage of 120 kV, a tube current of 80 mA, and 4.25 mm section collimation. Immediately after the CT, a PET scan was then obtained from the level of the head to the upper part of the legs at 3 minutes per bed position, usually 6–8 bed positions depending on the height of the patient. Reconstruction of the PET data was performed with the ordered set expectation maximization algorithm. A Xeleris workstation (GE Medical System) was used for evaluation of data obtained from both CT and PET.

### 2.3. Measurement of MTV and TLG

Two experienced nuclear medicine physicians analyzed all the images independently on Xeleris workstations to identify all definite cancer-related lesions. The lesions' locations were recorded to produce target volumes from PET/CT results. RT image, a free software program developed by the Department of Radiation Oncology and MIPS at Stanford University, was deployed to read all the primary CT and PET DICOM data. Each lesion was selected on PET images and segmented automatically using a 3D-area growing algorithm.

Four thresholds were chosen for delineation: (1) The absolute threshold (Th2.5) was calculated as SUVmax = 2.5 marking all voxels inside foci with SUVs > 2.5 as tumor tissue. (2) The relative threshold (Th20) was calculated as SUV= 20%  × SUVmax, meaning that all voxels inside the lesion with SUV higher than 20% of the SUVmax of the lesion were labeled as tumor tissue. (3) The relative threshold (Th40) was calculated as SUV= 40%  × SUVmax, indicating that all voxels inside the lesion with an SUV > 40% SUVmax of the lesion would be labeled as tumor tissue. (4) The relative background dependent threshold (Thbgd) was calculated as SUV = SUVbgd + 20% (SUVmax − SUVbgd), where SUVbgd was described as the mean SUVmax of the surrounding background of the ROI, that is, ten randomly outlined regions in the background around the lesion where their mean was SUVbgd. SUVmax ROI is indicated as the SUVmax of the lesion [22]. The volume and SUVmean of each lesion were calculated by the software. The MTV of each slice was then calculated by multiplying the area within the threshold margin. The sum of the MTVs of every lesion in a patient is the WBMTV. TLG is calculated by multiplying the MTV by the SUVmean [23]. The sum of the TLGs of each lesion is the whole-body TLG (WBTLG).

### 2.4. Statistical Analysis

We used SPSS statistical software version 17.0 (SPSS Inc., Chicago, IL, USA) for statistical analysis. We also used chi-square analysis and Fisher's exact test to determine differences in the lesion detection rate among the four threshold methods. The optimal SUV threshold method was then performed to calculate WBMTV and WBTLG. Receiver operating characteristics (ROC) curves were generated to assess the area under the curve (AUC) and the optimal cutoff value for WBMTV and WBTLG. The Kaplan-Meier method and the log-rank test were used to evaluate and compare survival rates. Overall survival (OS) and disease-free survival (DFS) were chosen as endpoints and were measured from the date of radiotherapy initiation to the date of death or recurrence. The prognostic significance of SUVmax, WBMTV, WBTLG, and other pathological variables for OS and DFS was assessed by Cox proportional hazards regression analysis.

## 3. Results

### 3.1. Patients

The characteristics of the patients are summarized in Table 1. Of 221 patients, recurrence and metastasis were confirmed in 28 patients. A total of 37 had died at the last follow-up. A total of 156 patients enjoyed DFS, which includes 90 normal and 66 residual cases. Five-year OS and mean survival time of all patients were 71.6% and 90.9 ± 0.6 months, respectively.

### 3.2. Comparison among the Four Thresholds

**Figure 1:**
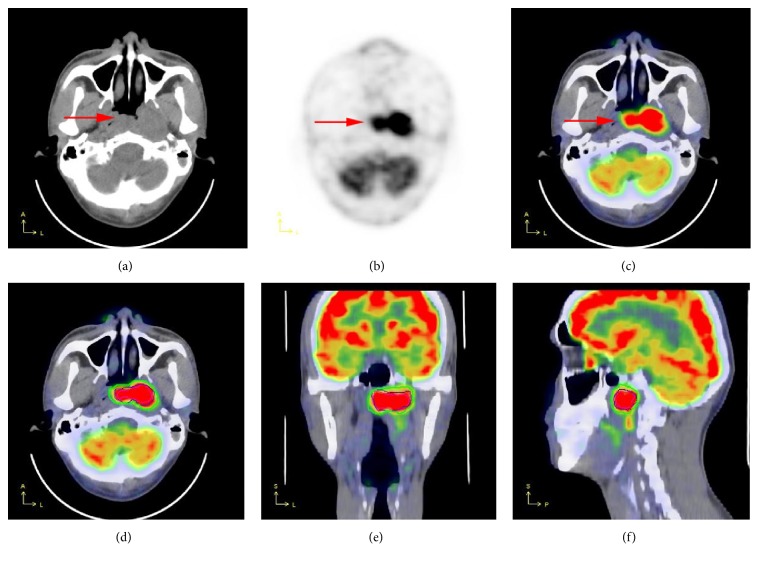
A 59-year-old male with NPC after comprehensive therapy. CT (a), PET (b), and fused (c) images show a mass lesion in nasopharyngeal posterior wall and left lateral wall (red arrow). Results of segmentation using the four described thresholds are shown on transaxial (d), coronal (e), and sagittal views (f). The light red, purple, blue, and green areas were segmented using SUVmax = 2.5, Th20, Th40, and Th_bgd_ thresholds, respectively.

The 221 patients had a total of 576 lesions (Table 2).  Figure 1 shows a case of NPC after comprehensive therapy, where the four threshold methods were used for segmentation. The detection rates of the threshold Th2.5, Th20, Th40, and Thbgd method were 65.6% (378/576), 80.2% (462/576), 71.5% (412/576), and 90.4% (521/576), respectively. The Thbgd threshold delineated significantly more lesions than the Th2.5, Th20, and Th40 methods (*P* < 0.001), while statistical differences could be seen among the Th2.5, Th20, and Th40 methods (*P* < 0.05). Th2.5, Th20, and Th40 thresholds mainly failed to segment lesions in the nasopharynx, the skull base, cervical lymph nodes, lung, abdomen, pelvis, liver, and bone; the Thbgd threshold mainly failed to delineate lesions in the lung, liver, and bone (Table 3).

### 3.3. WBMTV, WBTLG, and Prognostic Factors

**Figure 2:**
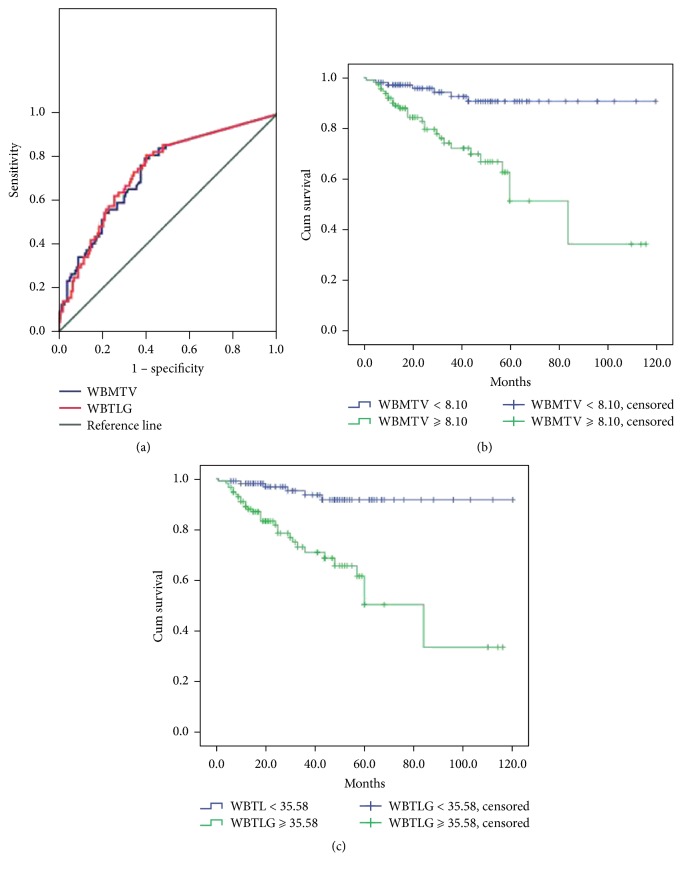
(a) The cutoff value of WBMTV and WBTLG was 8.10 and 35.58, respectively, from ROC analysis. (b) Five-year OS stratified by WBMTV (log-rank test, *P* < 0.001). (c) Five-year OS stratified by WBTLG (log-rank test, *P* < 0.001).

WBMTV and WBTLG were calculated according to the Thbgd threshold, which segmented most of the metastatic lesions. The mean values of SUVmax, WBMTV, and WBTLG were 5.57 ± 5.5 (range, 1.7–24.9), 15.2 ± 21.1 cm^3^ (range, 0–159.3 cm^3^), and 88.6 ± 127.9 (range, 0–900.9), respectively. From ROC curves, the cutoff values of WBMTV and WBTLG were 8.10 and 35.58 (Figure 2(a)), respectively. AUCs were 0.733 ± 0.037 and 0.736 ± 0.035, respectively. Patients with WBMTV < 8.10 had significantly better 5-year OS (90.7% versus 51.2%, *x*^2^ = 18.0, *P* < 0.001) than patients with a WBMTV ≥ 8.10. Patients with WBTLG < 35.58 had significantly better 5-year OS (91.7% versus 50.4%, *x*^2^ = 21.8, *P* < 0.001) than patients with a WBTLG ≥ 35.58 (Figures 2(b) and 2(c)). A Cox proportional hazards multivariate model of OS and DFS outcome was constructed to evaluate the age, gender, treatment, lesion number, SUVmax, WBMTV, and WBTLG as predictors of disease progression and survival. The results indicated that both WBTLG (RR, 1.002; *P* = 0.004) and age (RR, 1.046; *P* = 0.006) could be used to predict OS (Table 4). For DFS, WBTLG (RR, 1.003; *P* < 0.001) may have predictive relevance.

## 4. Discussion

The current standard treatment for NPC is radiotherapy. The treatment has effects such as edema, loss of tissue planes, fibrosis, mucositis, and scarring [24] resulting in serious complications to the patient and causing interference with the detection of local recurrent or persistent NPC. Accurate prediction of prognosis is vital for therapy planning. Identification of predictors associated with poor outcomes is of paramount importance before selecting appropriate candidates for such treatment modalities. ^18^F-FDG PET/CT is a noninvasive imaging modality which has the ability to visualize and quantify the glucose metabolism of malignancies including NPC. The purpose of our study was to investigate a number of PET-based functional indices and their relationship with the prognosis in NPC patients after comprehensive therapy. In our study, we found that the background threshold for segmentation of malignant lesions was much better than Th2.5, Th20, and Th40. WBTLG, a parameter that includes information on both tumor function and volume, was an important independent factor to predict the prognosis. However, WBMTV was found to be unrelated to the prognosis.

Tumor volume analysis was based on a set of thresholds. We selected four different thresholds for comparison in this study. Only 65.6% of lesions were segmented when the threshold was selected as SUV = 2.5. This is mainly because sometimes the background around the lesion was >2.5; while the computer was selecting the lesion area, the surrounding background might have been segmented as a lesion, hence making it harder in differentiating the tumor lesions from the surrounding normal tissue. Th20 and Th40 were rendered inadequate methods by some low-uptake lesions that could not be segmented (such as lung and liver) when the thresholds were lower than the surrounding background SUV. Significant FDG accumulation was always visible in some head-neck inflammatory areas resulting in excessive segmentation with the surrounding background. Using the background method to segment volume, the lesion detection rate reached 90.4%. The main advantage of this method is the inclusion of the surrounding background into the calculation of threshold, taking full account of the high FDG uptake area and the surrounding normal tissue. Yu et al. observed that the background method is able to predict primary lesion of esophageal cancer [22]. Liao et al. reported that the detection rate of lesions using the background method to calculate the threshold was significantly higher than that of using SUV 2.5 in epithelial-derived ovarian cancer patients after surgery [25].

Clinically, SUVmax, a metabolic index, is frequently used to assess tumor activity because it is an observer-independent measurement. Prior studies have documented the importance of SUVmax in predicting treatment response and survival in patients presenting with head and neck cancer and other types of malignancies [26, 27]. SUVmax, as a single voxel value, is susceptible to noise and therefore it may not accurately reproduce the overall tumor burden [28]. A previous study of NPC patients treated with radiotherapy or CCRT suggested that the SUVmax of the primary tumor was not an independent prognostic factor [29]. These findings are similar to those of an earlier published report, which observed that SUVmax of the primary tumor was not only a poor independent prognostic factor for survival but also a poor predictor of treatment response [30]. Therefore, adequate methods of identifying patients who are at risk and who may be candidates for aggressive initial treatment are crucial.

Chan et al. suggested that MTV is a prognostic factor in patients with head and neck cancer. Furthermore, they indicated that MTV appears to be an independent risk factor in advanced NPC patients [31]. Other studies focusing on lung cancer and lymphoma reported similar findings by showing that MTV is an accurate predictor of disease and death and thus is independent of other recognized prognostic factors [31]. A cutoff value of 30 cm^3^ for metabolic volume and 130 for metabolic index has been suggested to differentiate between favorable and unfavorable outcomes [32]. In our study, we discovered that 8.10 cm^3^ was the most discriminative cutoff for WBMTV. On further analysis, we found that patients with tumors with lower WBMTVs had higher 5-year OS than those patients with higher WBMTVs. However, there was no statistical significance in multivariate analysis due to the fact that all the patients in our study had received surgery or radiotherapy/chemotherapy resulting in inadequate statistical significance in the relationship with survival time. Thus, the current evidence highlights weaknesses of SUVmax and WBMTV.

TLG, which is derived from SUVmean and MTV, is regarded as an ideal metabolic variable in reflecting total tumor volume. TLG incorporates both anatomic (tumor volume) and biological (glucose metabolism) data, making it a more accurate predictor than either MTV or SUV [31]. An earlier report by Chan et al. suggested that TLG was more predictive than MTV for OS and DFS [31]. TLG has also been shown to be able to predict the response of epithelial-derived ovarian cancer patients to treatment [23]. Similar findings in the generation of prognosis by using diameter-SUVindex were found by Roedl et al. [33]. Both TLG and MTV have been found to be independent predictors of prognosis in groups of patients with malignant mesothelioma and oropharyngeal squamous cell carcinoma [34, 35]. Kim et al., in their study of 140 patients with diffuse large B cell lymphoma, showed that TLG with a 50% margin threshold was an independent prognostic factor for survival [36]. Consistent with this finding, we discovered that the value 35.58 was the most discriminative cutoff. A principal finding of this study is that WBTLG is strongly correlated with OS and DFS in patients with NPC after comprehensive therapy, and thus it is a better predictor of long-term survival than WBMTV and SUVmax alone. In our opinion, high WBTLG represents a tumor's aggressiveness.

Our study has some limitations. The low number of patients evaluated and the retrospective nature of our study restrict the magnitude at which our results can be applicable in a large-scale setting. The other limitation is the heterogeneity of the patients and different treatments protocols. The different time interval of PET acquisitions and variations of lesions locations were other added limitations. Furthermore, the possible risk factors, such as Epstein–Barr virus status and DNA level, were not included in the analysis due to limited cases with available data in our setting. Long-term prospective validation studies of large populations are necessary to confirm our findings.

### 4.1. Conclusion

By quantitatively analyzing ^18^F-FDG PET/CT images of NPC patients after comprehensive therapy, the SUV threshold background method had a significantly higher detection rate of metastatic lesions than using an SUV cutoff of 2.5, Th20, or Th40. WBTLG calculated from the Thbgd threshold could be used as an independent prognostic factor for patients with NPC postcomprehensive therapy. A prospective study with a large sample size is needed to further validate the reliability of this study.

## Figures and Tables

**Table 1:** Characteristics of 221 patients.

Characteristic	Number of patients	Constituent ratio (%)
*Age*		
Median	46 ± 12	
Range	17~75	
*Gender*		
Male	169	76.4
Female	52	23.5
*Treatment*		
Radiotherapy	89	40.3
Radiotherapy & chemotherapy	119	53.8
Surgery & radiotherapy & chemotherapy	10	4.5
Surgery & radiotherapy	1	0.5
Chemotherapy	1	0.5
Surgical	1	0.5
*Patient status*		
No evidence of disease	156	70.6
Alive with disease	28	12.7
Dead	37	16.7

**Table 2:** Location of definitive lesions in the included patients.

Position	Number
Nasopharynx	86
The skull nearby nasopharynx	92
Lymph nodes in neck/axilla/thorax	278
Pleura	3
Lung	36
Liver	20
Bone	61

Total	576

**Table 3:** Lesions unable to be segmented using different thresholds.

Location	Lesion numbers			
	Th2.5	Th20	Th40	Thbgd
Nasopharynx	31	25	28	2
The skull nearby nasopharynx	38	11	29	5
Lymph nodes in neck/axilla/thorax	58	28	41	15
Pleura	0	0	0	0
Lung	28	18	25	11
Liver	11	7	9	6
Bone	32	25	32	16

Total	198	114	164	55

**Table 4:** Cox proportional hazards regression analysis for OS of different factors.

Factors	*P*	HR	95% CI^*∗*^	
			Lower	Upper
Age	0.006	1.046	1.001	1.245
Gender	0.319	0.681	0.621	1.008
Treatment	0.523	1.078	0.801	1.107
Focus number	0.681	1.101	0.568	1.814
SUVmax	0.356	1.032	0.758	1.072
WBMTV	0.254	0.853	0.824	1.082
WBTLG	0.004	1.002	1.002	1.236

^*∗*^ *P* < 0.05. CI^*∗*^ indicates confidence interval.
